# Trends in the Development of Electronic Noses Based on Carbon Nanotubes Chemiresistors for Breathomics

**DOI:** 10.3390/nano12172992

**Published:** 2022-08-29

**Authors:** Sonia Freddi, Luigi Sangaletti

**Affiliations:** Surface Science and Spectroscopy Lab @ I-Lamp, Department of Mathematics and Physics, Università Cattolica del Sacro Cuore, Via della Garzetta, 48, 25133 Brescia, Italy

**Keywords:** carbon nanotubes, electronic nose, breathomics, gas sensing, breath analysis, chemiresistor

## Abstract

The remarkable potential of breath analysis in medical care and diagnosis, and the consequent development of electronic noses, is currently attracting the interest of the research community. This is mainly due to the possibility of applying the technique for early diagnosis, screening campaigns, or tracking the effectiveness of treatment. Carbon nanotubes (CNTs) are known to be good candidates for gas sensing, and they have been recently considered for the development of electronic noses. The present work has the aim of reviewing the available literature on the development of CNTs-based electronic noses for breath analysis applications, detailing the functionalization procedure used to prepare the sensors, the breath sampling techniques, the statistical analysis methods, the diseases under investigation, and the population studied. The review is divided in two main sections: one focusing on the e-noses completely based on CNTs and one reporting on the e-noses that feature sensors based on CNTs, along with sensors based on other materials. Finally, a classification is presented among studies that report on the e-nose capability to discriminate biomarkers, simulated breath, and animal or human breath.

## 1. Introduction

Disease diagnosis through exhaled breath is a technique that, even if in a rudimentary way, was known since the ancient Greeks, when for instance the fruity smell in the breath of children was used to diagnose diabetic ketoacidosis [1].

The most important impulse in the field of breath testing has been given in 1971 when Linus Pauling [2] reported on the identification of 250 compounds in the exhaled breath through gas chromatography, and researchers started to investigate the nature and the origin of those compounds in the breath. More recent studies identified up to 874 volatile organic compounds (VOCs) in human exhaled breath [3,4].

Nowadays, breathomics, or breath analysis, holds great promises to deliver rapid, non-invasive and low-cost diagnosis of diseases, playing a major role in screening campaigns for those pathologies, such as lung cancer, where an early diagnosis is crucial for the success of the treatment.

Breath analysis is based on the fact that the quantity and the quality of the VOCs present in the exhaled breath of healthy patients are different from the ones present in the exhaled breath of sick patients and that each pathology is related to the presence of one or few gas molecules (i.e., biomarkers) in a fixed quantity [4,5]. For instance, ammonia could be found in higher concentration in the exhaled breath of people affected by liver or kidney pathologies, with respect to the concentration found in healthy people [6].

The analysis of the exhaled breath could be conducted with two different ap-proaches. The first one is based on spectroscopic techniques, such as gas chromatography and mass spectroscopy. The second one benefits from the development of solid-state chemical sensors.

Indeed, an effective way to analyse breath samples with high sensitivity and accuracy is represented by gas chromatography-mass spectrometry (GC-MS). However, GC-MS is often time-consuming and expensive [7] and requires a well-trained technician to analyse the data, making their application in screening campaigns and clinical trials often not practical. Hence, gas sensors and electronic noses are regarded as better candidates to be used in this field and overcome the limitation of GC-MS, thanks to their capability to detect gas molecules in a non-invasive and rapid manner.

**Figure 1:**
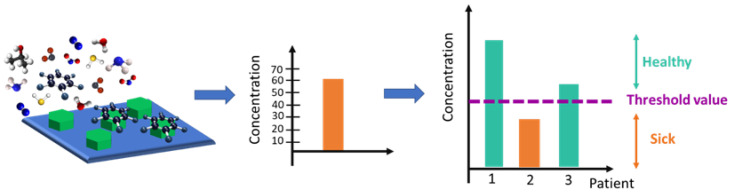
Schematic representation of the working principle of a highly selective sensor. The selective sensor has been properly functionalized with the aim of recognizing and detecting a specific gas-analyte among the interfering gases present in the exhaled breath. After the exposure, the sensor response is evaluated and linked to a specific concentration of the gas-analyte in the breath; finally, the obtained concentration value is compared with a threshold value, which leads to the discrimination between sick and healthy subjects.

The simplest way to use solid state gas sensors for breath analysis is to develop a very selective single sensor, able to detect only one analyte that is known to be a bi-omarker of the disease to be diagnosed. The biomarker is recognised by the sensor among a huge quantity of interfering gases, often thanks to an ad-hoc functionalization of the sensing layer. The discrimination between healthy and sick patients is conducted by (1) evaluating the response of the sensor, (2) estimating the concentration of the biomarker in the exhaled breath, and (3) comparing the measured value with a specific threshold: concentrations below (or above, depending on the specific biomarker) the threshold indicate healthy patients, vice versa they identify sick patients (see Figure 1). There are few examples of selective sensing of disease-related VOCs, including for instance the detection of nitric oxide [8], acetone [9,10], or ammonia [11,12]. Note that basically all the literature works dealing with those selective sensors involve tests conducted only in a laboratory, with synthetic gases, and did not use human exhaled breath or mixture of gases. Moreover, the selective sensor approach is quite limited, mainly due to the fact that there are almost no unique VOCs for a disease [13]. In addition, there are difficulties in the development of such sensitive and selective sensors. Indeed, one of the problems in the gas sensor field has always been dealing with interfering gases, which in exhaled breath are really numerous.

In order to avoid the selectivity problem, researchers started to look for new approaches based on solid state gas sensors as well. In particular, electronic noses, or sensors arrays, could play an important role in screening campaigns and early diagnosis.

**Figure 2:**
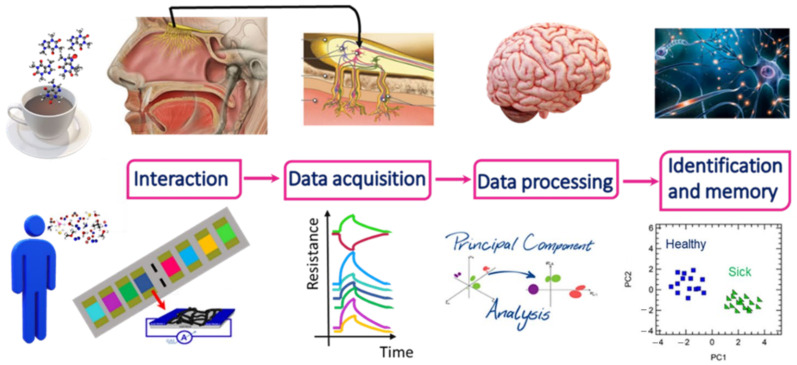
Schematic comparison of the working principle of the human nose and electronic nose.

Electronic noses have been designed with the aim to mimic the olfactive human system. As reported in Figure 2, the perception of odours (i.e., gas molecules) in the human nose is achieved through specific receptors located in the nasal cavity. In particular, gas molecules enter human nostrils and interact with the olfactory epithelium, which contains the nerve cells and their trigeminal endings. These trigeminal endings carry specific receptors that generate an electrochemical potential upon interaction with gas molecules. This potential variation is transmitted by neural axons to brain centres that read and interpret the incoming signals. After learning and training, the human brain can remember the signals related to different gases, which enable humans to recognize different odours and take memory of them.

Basically, an electronic nose consists of an array of *n* low selective sensors, which can therefore potentially respond to all, or part of, the VOCs/gas molecules present in the exhaled breath. Since the sensor response could not be directly linked to a unique selected VOC, statistical analysis methods based on pattern recognition and/or multivariate data analysis, such as principal component analysis (PCA), are required to properly analyse the data. Generally speaking, in the case of PCA the discrimination is achieved in a 2dimensional or 3dimensional space, where a breath sample is represented by a single point, which contains the information coming from all the n-sensors in the array. The points related to breath collected on healthy patients cluster in a different region of the space, as compared to the points collected on sick patients; it goes without saying that each disease generates a different clustering in a 2D/3D space.

In spite of being less selective to a specific VOC as compared to selective sensors, electronic noses are more versatile in detecting multi-component gas volumes in various atmospheres (often including those for which the arrays were not originally developed). Therefore, characteristics of all the sensors in the array, such as the detection limit or sensitivity, should be determined for targeted VOCs, as well as for the major interfering compounds, such as humidity.

The discrimination capability of the electronic nose is first tested by exposing the array to gases in the laboratory that are related to the VOCs one is interested in recognising. If the multivariate statistical analysis on the gas data results in good discrimination capability, breath samples can be tested. Otherwise, an improvement of the sensor array is needed in order to achieve the desired gas discrimination.

After the gas exposures test, a pathology should be selected and tests on the exhaled breath of patients that are healthy or already identified as affected by the selected pathology should be conducted. In this way, the discrimination capability will be assessed, and the resulting graph and data could be used as a “memory” in future screening campaigns for the selected pathology. This routine is called the training stage. After the training, data validation should occur.

It is important to mention that in principle all the pathologies could be investigated with breath analysis and electronic noses techniques, since the VOCs in the exhaled breath are generated by all the cells and organs in human beings and they are then transported to the lung through blood.

The challenges that breathomics is actually facing include the development of sensors that can detect very low concentrations of the biomarkers among interfering gases. Indeed, breath is mainly composed by oxygen, carbon dioxide, nitrogen and water vapour, with traces of VOCs. While VOCs such as acetone and isoprene could be found in a ppm range in the exhaled breath [14], most of the VOCs in the exhaled breath, for instance benzene, nitrogen dioxide, 2-propanol or toluene, are typically in a low-ppb range [15].

So far, breath tests are poorly used in clinical practice [1], but this technique holds great potential to be used in:

1. screening campaigns for those pathologies where an early diagnosis is crucial for the success of the treatment [16,17];
2. screening of patients for emerging diseases (for instance also COVID-19) [18] or high-risk populations [19];
3. low-cost diagnosis in developing countries [20];
4. evaluation and monitoring of therapy efficacy [21,22].

Finally, the use of electronic noses into the diagnostic routine could reduce the use of less efficient or invasive methods, leading to more rapid and convenient diagnoses, with financial savings in healthcare systems [5].

Currently, electronic noses are mainly based on conducting polymers or metal oxide sensing devices that still present several drawbacks, including a high operation temperature and therefore a high-power consumption, along with poor selectivity and poor stability [23,24]. However, carbon-based materials are considered as good candidates for gas sensors development, thanks to their outstanding properties.

Regarding carbon nanotubes, three are the main reasons why they are appealing systems to be used in gas sensors: (1) the huge surface to volume ratio, (2) their one-dimensional nature, which makes them very sensitive to tiny external perturbations and provides a very efficient charge transport along their tubular structure [25], and (3) their ability to operate at room temperature.

Although single CNTs-based sensors represent a huge class of single-gas sensors with remarkable sensing capability and many reviews have been already published, [25,26,27,28,29,30,31,32,33] only recently CNTs sensing layers have been implemented into electronic noses, therefore relatively few are the studies on electronic noses completely based on CNTs-sensors for breathomics and even less on arrays based on CNTs-sensors exposed to real human breath from healthy or sick people. Nevertheless, these works clearly demonstrate a huge potential for CNTs-based e-noses to be used in breathomics and can set the basis for using such e-noses in clinical trials. Up to now, only the work of Ellis et al. briefly introduce the topic of CNTs-based sensor arrays for breathomics [25], but a review which includes the last decade of studies is virtually missing. Therefore, the present work aims to fill this gap. This review will summarize the use of CNTs-based arrays in breathomics, detailing the type of sensing layers, the targeted disease, and the testing conditions.

First, a quick overview on the functionalization of the CNTs layers used for e-noses development will be given, then breath sampling procedures and statistical analysis will be introduced before disclosing details on the techniques reported in the 26 published works on CNTs-based chemoresistive electronic noses. Secondly, the diseases under investigation will be presented.

The review will then continue with two sections. In the Section 1, arrays completely based on CNTs-sensors will be described. In the Section 2, electronic noses featuring CNTs-based sensors and other material-based sensors will be detailed. Finally, a further classification will be considered, based on works that report test of electronic noses on disease biomarkers in the gas phase, works that used synthetic breath or non-human breath (i.e., rat breath), and works investigating the capability of the electronic noses to discriminate the human exhaled breath of different patient classes (healthy/sick or sick/sick with different pathologies).

**Figure 3:**
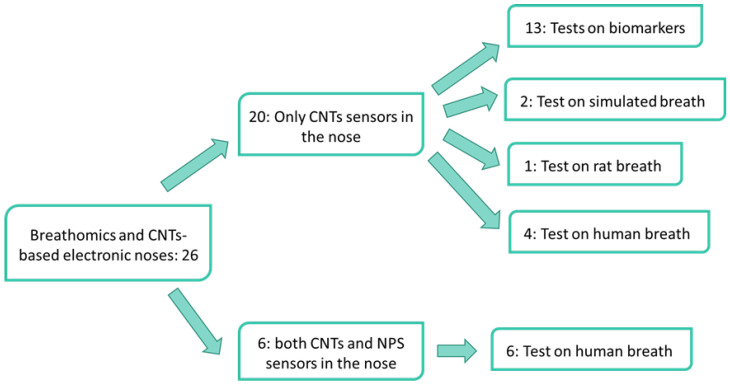
Flow diagram summarizing the structure of the present review. A total of 26 papers have been considered, 20 of them based on electronic noses featuring only CNTs sensors, whereas 6 papers reported on arrays based on CNTs and NPs sensors. A second classification is given by the type of analytes the papers are focused on: biomarkers, simulated breath, animal breath, or human breath.

Among the 26 articles found in literature reporting on CNTs-based electronic noses for breathomic applications, 20 of them present electronic noses completely based on CNTs-sensors [34,35,36,37,38,39,40,41,42,43,44,45,46,47,48,49,50,51,52,53], while 6 papers report on electronic noses that host both CNTs-sensors and NPs-based sensors [54,55,56,57,58,59]. All the electronic noses included in the latter group have been tested on the exhaled breath of patients, while only four electronic noses of the former group have been exposed to human breath [50,51,52,53], whereas 13 arrays have been tested only to target gases [34,35,36,37,38,39,40,41,42,43,44,45,46]. Tests on simulated exhaled breath have been performed by two electronic noses [47,48] and one array has been exposed to the breath of rats [49]. Figure 3 sums up the flow diagram on this classification.

## 2. Materials and Methods

### 2.1. CNTs Functionalization

The sensing properties of pristine CNTs sensors are themselves already remarkable, but functionalization can enhance their selectivity and sensitivity, increasing the discrimination capability of such sensors when they are implemented into an electronic nose.

Functionalization of carbon nanotubes could be either covalent, due to the formation of covalent bonds between the functionalization compound and CNTs surface, leading to a disruption of the sp^2^ system, or non-covalent, mainly due to Van der Waals or π-π interactions between CNTs and the functionalizing compound, which does not alter the carbon hybridization of the CNT structure.

When possible, covalent functionalization generally increases sensitivity more than a non-covalent approach [60,61], but non-covalent methods bear some advantages, including the often easier and low-cost functionalization procedure [62].

To date, only an article reports on covalent functionalization of CNTs-based sensors to develop an electronic nose [43], whereas the majority deal with CNTs modified via non-covalent techniques, including drop-casting of molecules or nanoparticles in solution [34,38,41,45,50], thermal evaporation [37,52,53], spray layer-by-layer deposition [35,36,39,40,42], plasma post-discharge [46], or spin coating [51].

### 2.2. Breath Sampling Procedure

Two different components of the exhaled breath can be collected and analysed: alveolar breath and total breath. Alveolar breath is the portion of the exhaled breath involved in the air-blood exchange and therefore contains the highest concentration of volatile organic compounds coming from the metabolic activity of the cells, and very low contaminations [63]. Total breath includes the alveolar breath and the so called ‘dead breath’, the first portion of the exhaled breath that is not involved in the air-blood exchange and contains a higher amount of contamination.

**Figure 4:**
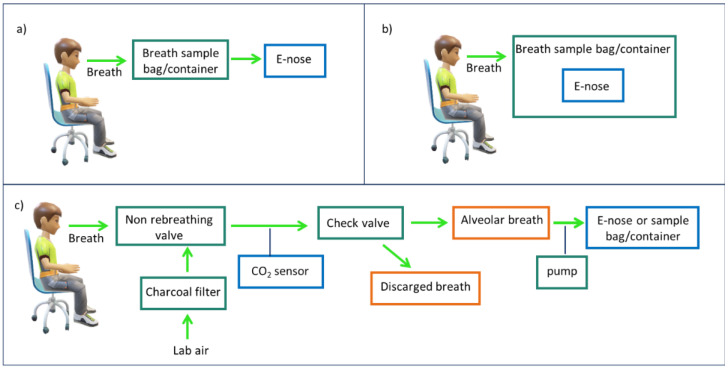
Schematic comparison between total breath (**a**,**b**) and alveolar breath (**c**) sampling. Total breath could be collected in a bag/container and then released on the electronic nose (**a**) or the electronic nose could be placed inside the container, as proposed for instance by [52] (**b**). Alveolar breath sampling requires a more complex apparatus, involving mainly a charcoal filter, a CO_2_ sensor, and a pump. Alveolar breath is mainly stored in sample bags or containers but can be also fluxed directly on the e-nose, as proposed in ref [49].

Dealing with alveolar breath allows for a reduction of the contamination and a more precise analysis of the volatile compounds. Nevertheless, collecting total breath is simpler and does not require additional equipment [64]. Figure 4 reports a schematic representation on alveolar (c) vs. total breath (a), (b) sampling: it is clear that alveolar sampling requires a more sophisticated apparatus.

The most common set up for collecting alveolar breath involves a charcoal filter, which assures no VOCs contamination from the room air, and a CO_2_ sensor [65,66,67,68]. Indeed, CO_2_ concentration is quite low in the dead space breath, while it increases considerably in alveolar breath. A pump allows for the alveolar breath collection, and the discharge of the dead space breath portion. Alveolar breath can be sent directly into the e-nose or into a sampling bag.

**Figure 5:**
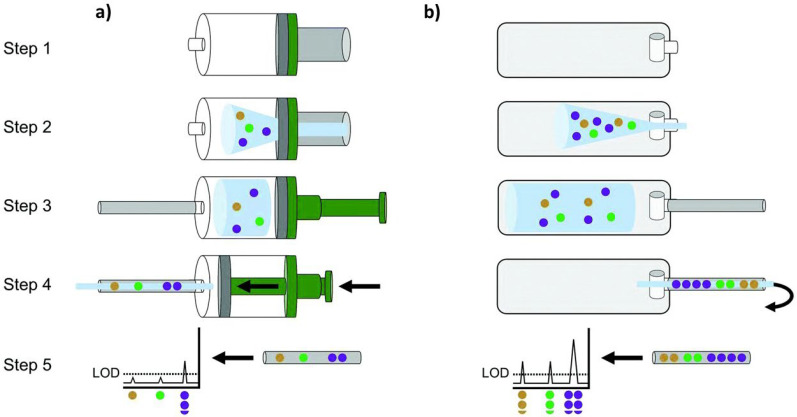
Comparison between paediatric (**a**) alveolar breath collection (via Bio-VOC™) and (**b**) total breath sampling (plastic bag). Step 1: the empty collection device. Step 2: patient exhales into the collection device. Different colour dots represent different VOCs. Step 3: Sorbent tube is affixed to the collection device. Step 4: Breath volume driven through the sorbent tube by depressing plunger or using an air pump. VOCs are captured on the sorbent tube. Step 5: VOCs are released from the sorbent tube by thermal desorption and measured by GC/MS. LOD: Limit of detection. Overall, the total breath sampling bag collects a larger volume of breath (Step 3) leading to a greater quantity of breath VOCs captured. This in turn is reflected as higher signals by GC/MS, including multiple breath VOCs that were undetectable via the alveolar breath collector Bio-VOC™ (Step 5). Adapted from ref [69] with permission from the Royal Society of Chemistry.

Finally, Berna et al. [69] demonstrated how sampling bags collecting total exhaled breath are better suited to search for biomarkers and volatiles in children, as evidenced by superior breath VOCs detection. (Figure 5).

Exhaled breath could be collected directly into an analytical instrument for an online analysis or stored in a container for offline analysis. The main containers for storage are Tedlar, Mylar and Nalophan bags, Flexfoil, glass vials, metal canisters, Teflon (PTFE) bags, and thermal desorption tubes [70].

Tedlar bags are the mostly used, although polar compounds could stick on the surface [70,71] and they are altered by sulfur-based VOCs [72]. Better performances are registered using silanized metal containers and thermal desorption tubes, but they are more difficult to use than plastic containers and much more expensive [14]. Nalophan and PTFE bags are not suggested for storage of VOCs containing sulfur compounds [72].

Comparing Tedlar and Mylar bags, contamination of the samples after 20 h is higher for the former [73].

Measurements performed with CNTs-based e-noses have been mainly conducted offline on alveolar exhaled breath [49,50,51,54,55,56,57,58], and mainly used Mylar bags [50,54,57,58].

Online measurements have been conducted only in ref [49,52,53], and total breath has been analysed only in [52], [53], and [57]. Of note, the PTFE bag used by Freddi et al. [52] and Drera et al. [53] was aimed at both collecting the exhaled breath and hosting the e-nose during the measurements..

Table 1 summarizes the method (online/offline), type of breath (alveolar/total), and breath container for the analysis with CNTs-based e-nose.

### 2.3. Statistical Data Analysis

PCA, as already mentioned, is the most used statistical tool for electronic noses data analysis. It basically relies on the possibility of obtaining valuable information from a data set where a great variability is observed.

In detail, PCA is an unsupervised multivariate statistical method which represents the main linear technique for dimensionality reduction, redistributing the total response in a set of principal orthogonal components (PCs); PC1 represents the dimension with the greatest variance, PC2 the second greatest variance, and so on. In general, only the first relevant PCs need to be taken into account. When only two relevant PCs, obtained by plotting PC1 as a function of PC2, are present in the resulting space, the response data to each exposure could cluster in separate regions of the 2D PC1 vs. PC2 plot and enable the discrimination of different target molecules or groups of subjects, who share similar breath-prints, belonging to different classes, e.g., healthy subjects vs. sick subjects. If necessary, additional coordinates can be considered. For instance, if PC3 is also considered, the clustering is studied in a 3-D space rather than in a 2-D space. Mathematically, each principal component is a standardized linear combination of the original variables (combined both positively and negatively) that enables a holistic interpretation of a multivariate phenomenon [74,75].

PCA is very useful in identifying data memberships, but one of its limitations lies in the fact that it is not a quantitative approach and therefore other statistical methods need to be coupled with PCA in order to obtain accuracy in the class prediction. One of these methods is the Support-Vector Machine (SVM). Indeed, it allows for the definition of a cluster boundary, by finding a data subgroup with the minimal interclass distance. Furthermore, in addition to the identification of the best class border, SVM also allows for the calculation of identification probability, i.e., for the definition of a continuous function, which assigns a likelihood for each data point to belong to a certain class [76]. To sum up, carrying out PCA coupled with SVM on a test dataset allows for the building of a model, whereas the addition of a testing dataset validates the model. PCA + SVM can therefore predict the class to which the data belong, providing an identification performance index (the ratio of the correctly labelled data) and an overall accuracy index, for proper data classification. In a best-case scenario, both these values should be close to 1.

PCA is not the only statistical method used to analyse data from an electronic nose. Among other statistical methods, it is worth to mention linear discriminant analysis (LDA) and discriminant factor analysis (DFA). Both LDA and DFA are supervised statistical methods for reducing the dataset dimensionality.

DFA is a linear method that is supplied with the classification information regarding every measurement in the training set. DFA finds new orthogonal axes (canonical variables) as a linear combination of the input variables, minimizing the variance within each class and maximizing the variance between classes [77].

DFA is in principle very similar to PCA; the major difference lies in the fact that PCA is an unsupervised method, therefore it calculates the best discriminating components without an a priori knowledge on the classes, whereas discriminant analysis, being a supervised method calculates the best discriminating components for classes that have been already defined by the user [78]. Furthermore, DFA is a predictive statistical method. The most common validation technique used in combination with DFA is the leave-one-out cross-validation, which provides the number of true positive (TP), true negative (TN), false positive (FP) and false negative (FN) predictions. In detail, given n measurements, the DFA model is computed using n-1 training vectors. The last vector, called the validation vector, is left out during the training phase, and then projected onto the model to provide information on the classification results. Finally, the goodness of the model could be evaluated defining the following parameters:

Sensitivity: TP/(TP + FN)

Specificity: TN/(TN + FP)

Accuracy: (TP + TN)/total samples

LDA, unlike PCA and DFA, deals with the covariance of the data matrix only, taking into account the class properties of each sample and assuming that each class has identical covariance. Optimal clustering among different classes or groups is obtained by maximizing the distance between classes and minimizing the scattering of the data inside each class, resulting in a reduction of the problem dimensionality, and obtaining a new space generated by new coordinates, called discriminant factors. The number of classes minus one gives the number of the new coordinates [79]. LDA gives a better separation among different classes then PCA, allowing for an easier classification. However, being a supervised method, it requires the a priori knowledge of each individual class, making it less suitable for prediction models.

More recently, artificial neural network (ANN) has also been considered to analyse the data obtained from CNTs-based electronic noses [80], especially when they are applied in clinical trials and a large number of data are collected [16,81,82,83].

**Figure 6:**
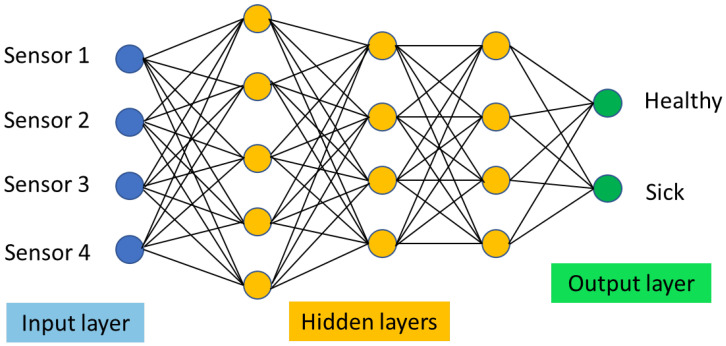
Simplified scheme of an ANN system architecture. In this example the e-nose is composed with 4 sensors.

ANN uses machine learning algorithms to mimic the way the human brain, through its neural system, analyses and recognizes information. In detail, ANN includes several nodes, or neurons, which are grouped in different layers, connected to one other with different weights. In particular, the data collected from the n-sensor array of the e-nose defines the n-input layer, while the classification in healthy or sick patients represents the output layer. All the nodes between the input and the output are called hidden layers and they extract information from the input layer in order to feed the output layer for the required classification. The number of the hidden layers usually ranges between the number of the nodes forming the input and the number of the nodes of the output layer. Figure 6 reports a typical scheme of an ANN.

The ANN needs to be, at first, trained to recognise the characteristic pattern of a healthy or sick patient. Therefore, a first exposure of the exhaled breath of patients with a sick or healthy diagnosis should be conducted and this dataset will be identified as the training data set.

Once the ANN learns to recognise the features identifying a sick or healthy patient in the training data set, the ANN needs to be validated, using a second dataset, i.e., the validation dataset, which contains data from patients sick or healthy, but where this last information will not be included in the dataset. If the ANN has been correctly trained, the output on the validation dataset should match with the known health status of the patients, with quite a high accuracy.

Once the ANN is validated, the ANN can be used to define the unknown healthy status of patients.

Data coming from electronic noses based on CNTs are mainly analysed through PCA or DFA. The former is the most used analysis in studies based on the discrimination of biomarkers, whereas the latter is mostly used to analyse data obtained from e-noses exposed to human exhaled breath, as will be detailed in the results and discussion part.

### 2.4. Targeted Diseases

The most common diseases investigated with commercially available electronic noses and in clinical trials are in general, diseases related to the respiratory system and lung [84]. The most studied pathologies are: airway obstructions diseases (including asthma, chronic obstructive pulmonary disease, and obstructive sleep apnoea syndrome), respiratory infections (such as ventilator-associated pneumonia and ear-nose-throat infections), inflammatory diseases (comprising acute respiratory distress syndrome, sarcoidosis, inflammatory bowel disease and inflammatory answer to ozone), and cancer (lung cancer, colorectal cancer, prostate cancer, and malignant pleural mesothelioma) [85].

**Figure 7:**
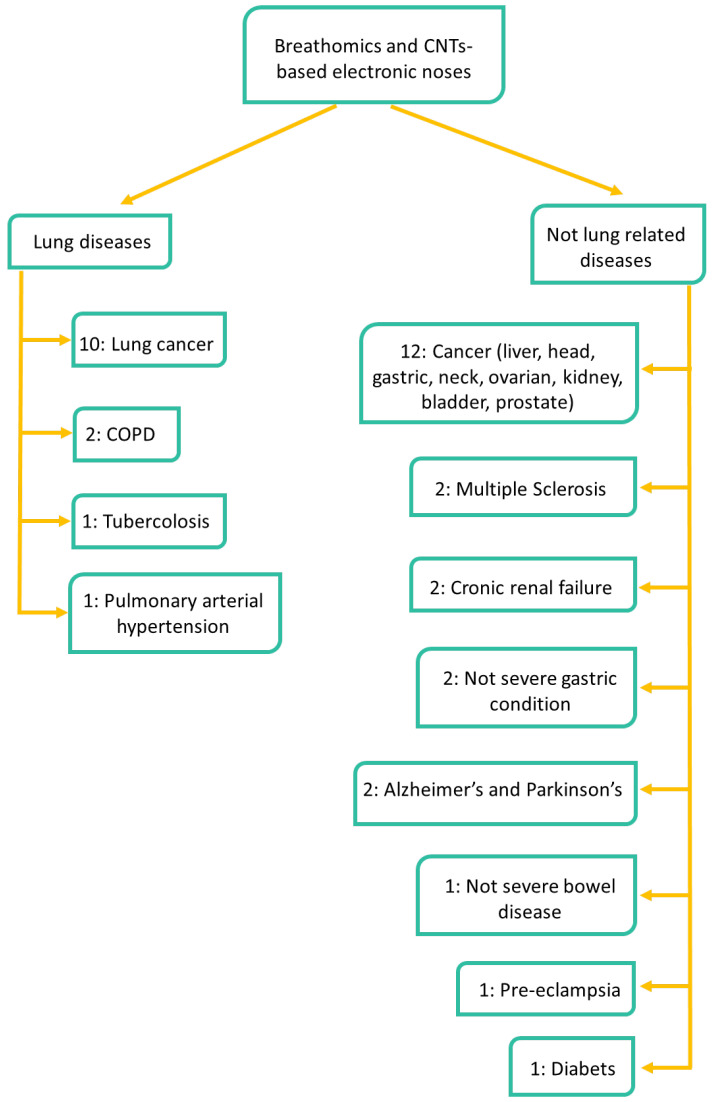
Flow diagram summarizing the pathologies investigated by the 26 articles reporting on CNTs-based electronic noses. Of note, the sum of the papers dealing with all pathologies is larger than 26, since some articles investigate more than one disease.

Figure 7 shows a flow diagram reporting the diseases targeted with CNTs-based electronic noses. Lung related pathologies are the most studied and therefore we decided to split the addressed pathologies in two classes: lung and non-lung related diseases. Regarding the former class, the most studied pathology is lung cancer (10 articles), followed by COPD (2 articles), and finally tuberculosis, and pulmonary arterial hypertension (1 article).

As concerns the latter, cancer is the most investigated pathology (12 articles), including liver, head and neck, ovarian, bladder, colorectal, kidney, as well as gastric and prostate cancer. Multiple sclerosis, chronic renal failure, non-severe gastric conditions, Alzheimer’s, and Parkinson’s diseases are investigated in 2 articles each. Finally, one article focuses on non-severe bowel condition (including Crohn’s disease (CD), ulcerative colitis (UC), irritable bowel syndrome (IBS)), diabetes and pre-eclampsia (PET). Of note, the sum of the papers dealing with all pathologies is larger than 26, since some studies address more than one disease.

### 2.5. Study Population

Table 2 reports the characteristics of the volunteers enrolled in each study reported in the works considered in the present review and it details the gender, average age, and the smoking habits, the latter being a risk factor for many pathologies. For almost every work it is clear that there is a balance in gender, average age and smoking habits in the studied population. When there is no balance, authors often prove that these factors do not influence the discrimination capability of the electronic nose [52,53].

## 3. Results

### 3.1. Electronic Noses Based on CNTs-Sensors

#### 3.1.1. Biomarkers Discrimination

The assessment of the capability to detect and discriminate biomarkers is crucial for each developed electronic nose and all the papers presented in this review tested this capability. As reported in the flow diagram (Figure 3), 13 studies, after focusing on the biomarker discrimination, conducted tests on simulated exhaled breath, exhaled human breath or animal breath, while the remaining 13 only assessed the capability of the e-noses to discriminate biomarkers proving their potentialities for breathomics applications.

The most investigated biomarkers are lung cancer biomarkers, i.e., ethanol, methanol, propanol, acetone, butanone, toluene, benzene, cyclohexane, pentene, dichloromethane, heptane, isopropanol, and tetrahydrofuran [35,36,39,40,41,42,45].

Six articles do not identify a specific target disease, but they generally prove the capability of the electronic nose to discriminate biomarkers related to several diseases, including liver and kidney failure (ammonia) [34,37], COPD (nitrogen dioxide) [34,46], diabetes (acetone) [34,36,38], asthma (hydrogen sulfide) [34], or again lung cancer [34,38,43,44,46].

The number of the sensors in the electronic noses so far considered varies from 3 to 10. The materials used for the CNTs functionalization can be divided in three classes: nanoparticles, including metal and metal oxides NPs and organic NPs [34,36,37,46], polymers [35,40,41,42], and organic compounds [38,39,43,44,45].

PCA has been used to analyse the data in almost all articles, whereas only Leighrib et al. [46] used LDA and Zilberman et al. [44] analysed the data through DFA.

Table 3 summarizes the number and type of the sensors composing the electronic nose based on CNTs, number and type of biomarkers investigated, target disease, and type of data analysis.

Human breath can reach 100% of relative humidity value, and therefore water molecules are regarded as the most important interfering gas in breathomics. Consequently, in order to gain information on the electronic nose behaviour in the presence of high humidity, exposures to water molecules are often performed, in addition to the exposures to the target biomarkers [34,36,39,40,41,42,45]. Furthermore, it was also suggested to perform exposures to the target gas at different humidity values [36,44].

Finally, a method to account for the effect of relative humidity when performing all kinds of gas exposures has been recently proposed: the response of a relative humidity sensor was added to the data feeding the statistical analysis (in the specific case, PCA), and a better discrimination of VOCs could be achieved [34].

One of the limits of testing single biomarkers lies in the fact the discrimination capability of the array is assessed in a condition which is not exactly the same as for tests on exhaled breath, not only for the presence of high humidity, but also because in the exhaled breath the VOCs are mixed together. Some studies tried to overcome this limitation, conducting exposures to mixture of two or more gases and obtaining good discrimination capability [34,35,37].

#### 3.1.2. Simulated Breath

**Figure 8:**
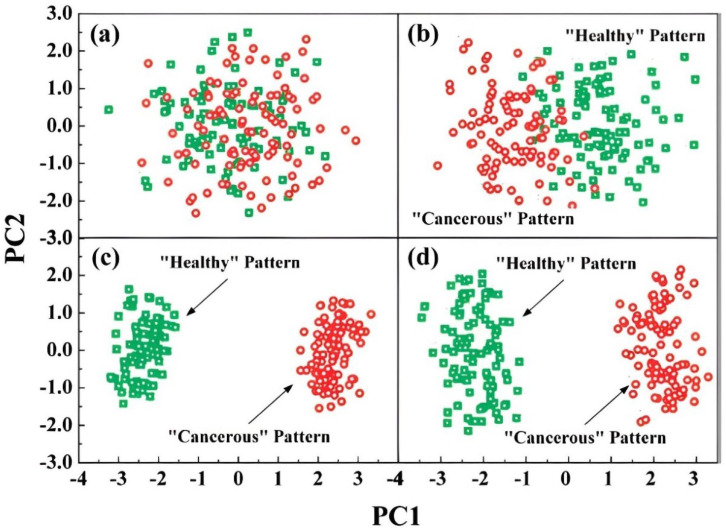
Principal components score plots upon exposure to simulated “healthy” and “cancerous” patterns at (**a**) 80% RH; (**b**) 10% RH; (**c**) 1% RH; and at (**d**) 80% RH and preconcentration of 50 times. Reprinted with permission from [47]. Copyright (2008) American Chemical Society.

A further step in the application of CNTs-based electronic noses in breathomics is achieved when researchers started to test the array to synthetic breath simulating healthy or sick patients’ breath. The first work analysing the behaviour of a chemiresistor CNTs-based e-nose to simulated breath has been published in 2008 by Peng et al. [47]. The array includes 10 sensors based on CNT coated with nonpolymeric organic materials, and the studies have been conducted in three phases: (1) At first, alveolar exhaled breath has been collected from lung cancer patients and analysed by GC-MS in order to identify the lung cancer biomarkers; (2) Exposures to the identified biomarkers have been performed and PCA demonstrates that the e-nose can clearly discriminate the lung cancer biomarkers in a 3D PC-space; (3) The e-nose has been exposed to healthy and cancerous simulated breath and analysed with PCA. A detailed analysis on the influence of relative humidity (RH) of the simulated breath has been performed as well. In particular, it was shown that decreasing the humidity content at first from 80% to 10% and then from 10% to 5–1%, a significant improvement of discrimination between healthy and cancerous patterns in a 2D-PCA space could be achieved. As an alternative to the decrease of the RH value, the authors also proposed to preconcentrate the lung cancer VOCs, while maintaining the RH at 80%, obtaining an excellent discrimination between healthy and cancerous patterns, comparable to the results obtained with the lower RH value (see Figure 8). This work [47] represents a proof of concept in the feasibility of using CNTs-based arrays in breathomics and opened up the possibility of carrying out tests on exhaled breath.

A decade after the publication of the work of Peng et al. [47], a second study exploring simulated breath has been published by Park et al. [48]. The array includes nine ionic liquid-SWCNTs sensors, and it has been tested at first against five tuberculosis biomarkers and their mixture, and secondly against simulated sick breath (three samples), healthy exhaled breath (three samples) and toluene (three samples), disclosing remarkable discrimination capability of the array when performing PCA on both datasets.

In spite of the limited data set, the array could be regarded as promising for breathomics, since it has great potential for miniaturization and easy integration in electronics, as well as an easy and solvent-free fabrication.

#### 3.1.3. Non-Human Breath

**Figure 9:**
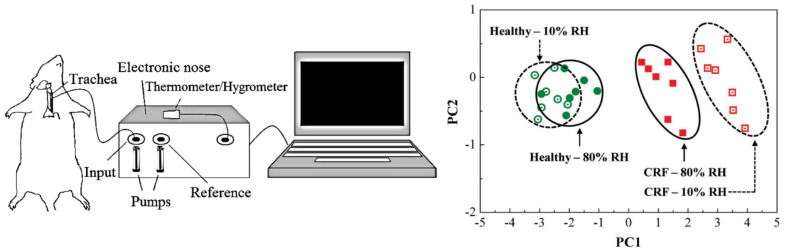
(**Left side**), schematic illustration of the experimental system used to collect breath from rats; (**right side**), PCA results on the response collected by the 10 sensors array upon exposure to the breath of healthy and CRF rats (with 80% RH), before and after dehumidifying the breath (80% RH vs. 10% RH). Better discrimination is obtained for lower humidity value. Reproduced with permission from [49]. Copyright (2009) American Chemical Society.

The analysis of the VOCs in animals’ breath is of great importance not only in veterinary care [87], but also for medicine. The research of biomarkers in animals and the understanding of their metabolic origin could be transferred to human beings, disclosing important information on VOCs formation and their link to particular diseases [88]. So far, only Haick et al. [49] reports on an electronic nose based on CNTs tested on non-human breath, namely, rat breath. The array includes 10 chemiresistive sensors based on a random network of SWCNTs coated with organic materials. The work focuses on the discrimination of breath from rats with chronic renal failure (CRF) and healthy control rats, involving 14 total rats (7 CRF, 7 healthy). To collect breath samples, a tube has been connected to the rats’ trachea (see scheme in Figure 9—left side).

The tube is divided in two parts: an outlet, which directly connected the rat to the electronic nose for breath analysis, and an inlet, which allows the rat to inhale the room air. PCA has been performed to analyse the data collected by the electronic nose, revealing excellent discrimination capability of the array, especially when dealing with low humidity (see Figure 9—right side). Authors also claim that using a rat to carry out the study rather than human breath allows for a better control of the experimental conditions and therefore yields more reliable and valid results.

#### 3.1.4. Human Exhaled Breath

To date, only four works report on electronic noses based on CNTs and tested on exhaled breath. Table 4 summarizes the number and type of sensors in the electronic nose based on CNTs, target disease, type of data analysis, and number of patients involved in the study.

The first paper on an electronic nose based on CNTs and tested to exhaled breath has been published in 2011 by Ionescu et al. [50]. The array is composed of four bilayers of polycyclic aromatic hydrocarbons and SWCNT. Following successful tests on nine biomarkers, the array has been tested to the alveolar breath of 51 volunteers, 17 healthy and 34 affected by multiple sclerosis (MS). For each of the four sensors in the array, three parameters have been evaluated during the gas exposures: the resistance change of sensor at the end of the exposure and at the middle, and the area under the response curve. DFA analysis on the data set demonstrate the capability of the array to discriminate between healthy and MS patients with an accuracy of 80.4% (85.3% of sensitivity and 70.6% of specificity).

An array prepared with eight sensors based on polymer/CNTs composites have been prepared and tested on the exhaled breath of five patients affected by hepatocellular carcinoma and five healthy control patients [51]. PCA has been performed to analyse the data and the results plot in a space generated by PC3 vs. PC1 show good discrimination capability between healthy and sick patients. The dataset is quite limited and only male patients have been involved in the study. Furthermore, no comment is reported by the authors on the age difference between healthy patients (35–60 years) and patients diagnosed with liver cancer (60–69 years).

Finally, two recent works investigate chronic obstructive pulmonary disease (COPD) with an electronic nosed composed of eight sensors based on SWCNTs functionalized with organic molecules [52,53].

COPD is a quite aggressive airway chronic inflammatory disease, which progresses over time, and it is not reversible. It has been reported by the World Health Organization that COPD is actually the fourth leading cause of death, and it will become the third by 2030 [89]. Nevertheless, this pathology is still quite not well known, and there are often delays in its diagnosis [90]. The use of electronic noses for a first screening campaign could improve the diagnosis rate, speed up the beginning of the therapeutic treatments, show the progress of the disease, and increase the life expectancy of the patients.

Freddi et al. [52], on the basis of the good ability of the array to discriminate through PCA selected target gas molecules including the COPD biomarkers, tested the developed array on 42 samples of human exhaled breath collected from 21 patients. The e-nose was able to distinguish healthy subjects from COPD subjects and this classification ability has been further improved by selecting the most responsive sensors to nitrogen dioxide, which has been proposed as a biomarker of COPD. Finally, the authors also prove that age, gender, and smoking habits of the volunteers do not influence the discrimination capability of the array, as well as relative humidity of the exhaled breath.

The same group conducted with the equipment of Ref [52] a second measurement campaign involving at first, 11 volunteers (7 COPD, 4 healthy), then, 50 subjects (30 COPD, 20 healthy) [53]. A combination of PCA, SVM, and LDA methods on 52 breath samples collected from 11 volunteers show that the electronic nose can be trained to clearly discriminate healthy from COPD subjects, in spite of the relatively limited dataset, with an accuracy index above 90% and 97%, for PCA and LDA, respectively. They also demonstrate that increasing the dataset (up to 130 samples from 50 volunteers), the discrimination capability of the array is maintained.

**Figure 10:**
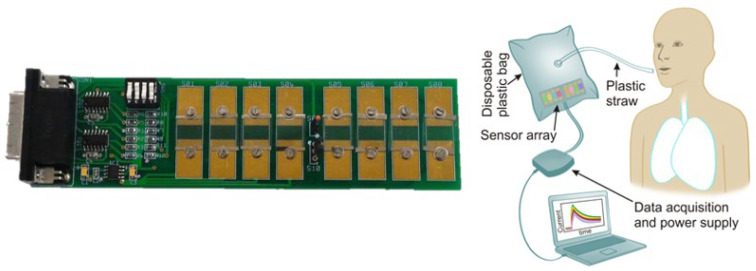
(**Left side**), picture of the eight-sensor array reported by [52,53]; reproduced with permission from [52]. (**Right side**), schematic representation of the breath sampling collection method reported by [52,53]: a disposable polytetrafluoroethylene (PTFE) bag contains the sensor array. After inserting the sensing unit, the bag was zipped on the side where the cable connected the unit to the data logger. Subjects inhaled to maximal inspiration and inflated the PTFE bag through a plastic straw. After the bag was inflated, the straw was extracted, and the bag was properly sealed to preserve the integrity of the sample. The sensor array inside the bag was exposed to exhaled breath for about 180 s to let all sensors fully interact with the target molecules. Then, the bags were opened, and the sensor array was cleaned with dry air before the next measurement to allow the sensors to recover. Reproduced from Ref. [53] with permission from the Royal Society of Chemistry.

The main strength of the studies by both Freddi et al. [52] and Drera et al. [53] lies in the fact that the breath samples collection is quite easy (a description can be found in Figure 10), and the robustness of their approach supports potential applications of the developed electronic nose in harsh environments where control over strict sample collection protocols may not be feasible.

The data presented in the four works [50,51,52,53] are only a proof of concept and of course, larger clinical studies are required to formally assess and validate the classification performance of CNTs-based electronic noses, using larger training and testing datasets, and to assess their potential clinical utility in the diagnosis and phenotyping of individuals with COPD, liver cancer and multiple sclerosis. Nevertheless, these results open the possibility to such clinical trial studies and to a future application of electronic noses for screening campaigns. In particular, the access to larger training and testing datasets could also open the possibility of using other data analysis tools, such as deep neural network.

### 3.2. Electronic Noses Based on CNTs-Sensors and Gold NPs-Sensors

Works in refs [54,55,56,57,58,59] have some common points. All of them combined the use of gas chromatography/mass spectroscopy (GC-MS) with the use of a nanostructured electronic noses, the former to identify the biomarkers and the latter to discriminate among different groups of patients. Furthermore, all the electronic noses host sensors based on organically stabilized spherical gold NPs and sensors based on SWCNTs capped with polycyclic aromatic hydrocarbons, in a different ratio (as reported in Table 5). Finally, all the electronic noses have been tested on alveolar exhaled breath and the data analysis has been conducted with the discriminant factor analysis (DFA).

Table 5 summarizes the number of the sensors in the electronic nose, the target disease and the number of patients tested.

**Figure 11:**
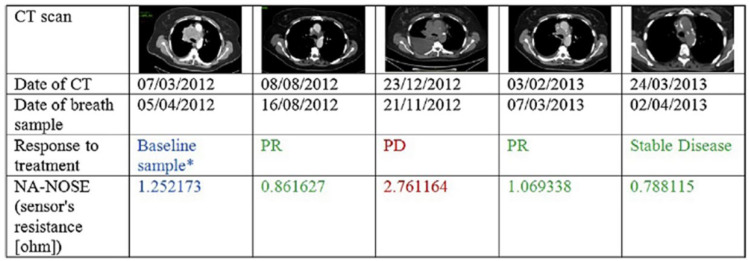
Example of consecutive CT scans for a SCLC patient in the study with the corresponding breath samples and electronic nose (NA-NOSE)’s resistance. * Before initiation of treatment. CT, computerized tomography; SCLC, small cell lung cancer; PD, progressive disease; PR, partial response. Reproduced from [54], copyright (2016), with permission from Elsevier.

Nardi-Agmon et al. [54] investigated the response to treatment in advanced lung cancer (LC) patients. Currently the accepted standard to monitor treatment efficacy in lung cancer are computerized tomography (CT) scans, but the time intervals between consecutive CT scans is often too long to allow for an early identification of treatment failure, therefore Nardi-Agmon et al. proposed the use of an electronic nose as an alternative and rapid tool to monitor the treatment efficacy. A total of 143 breath samples were collected from 39 patients with LC at different stages of the treatment: BL (baseline-before treatment), PR (partial response), stable disease, DC (disease control), and PD (progressive disease). The study has been conducted in two steps. First, DFA has been performed on the breath of BL and PR or stable disease breath. Secondly, PR/stable disease breath has been compared to PD breath. The first step assesses the capability of the array to discriminate BL patients from PR/stable disease patients, with 93% sensitivity and 85% specificity. The second step discriminates PD from PR/stable disease patients with 100% specificity but only 28% sensitivity, disclosing the potentiality of the array of detecting the lack of success in treatment. Figure 11 shows a comparison between consecutive CT scans for a SCLC patient and the corresponding breath sampling with the electronic nose, proving the potential capability of the latter of discriminating the success of the treatment.

To sum up, the results show that an oncologist can assess if the patient did not respond to the treatment (PD) with high accuracy using an electronic nose to speed up the recognition of the treatment failure and improve the patient care.

Gastric diseases, including precancerous gastric lesions, gastric cancer, and benign gastric conditions have been considered by both Amal et al. [55] and Xu et al. [56]. In particular, an early detection of gastric cancer (GC) and the related precancerous lesions by an electronic nose could provide a rapid diagnostic tool to reduce both cancer mortality and incidence.

A number of 968 breath samples have been collected from 484 patients with different gastric conditions by [55] and DFA results demonstrate that the nanoarray is able to discriminate between GC patients and the control groups (containing patients with peptic ulcer disease (PUD), precancerous lesion and dysplasia) with 73% sensitivity, 98% specificity, and 92% accuracy.

Xu et al. [56] proposed the use of an electronic nose as a rapid and alternative tool to upper digestive endoscopy with biopsy and histopathological evaluation of the biopsy material for GC diagnosis. Alveolar exhaled breath from 130 sick patients, i.e., 37 GC patients, 32 patients with ulcers, and 61 with less severe conditions, has been analysed with the DFA method. The results show excellent capability (sensitivity always equal or higher than 84%, specificity equal or higher than 87%) of the e-nose to discriminate between the following subclasses: GC vs. benign gastric conditions, early-stage GC vs. late-stage GC, ulcer vs. less sever conditions.

Head and neck squamous cell carcinoma (HNSCC) has been investigated by [57]. Authors assessed that to date, there is not an adequate method for an early detection or screening of the high-risk population of HNSCC. Therefore, the use of an electronic nose can establish the basis for a diagnostic/screening tool for such type of carcinoma.

**Figure 12:**
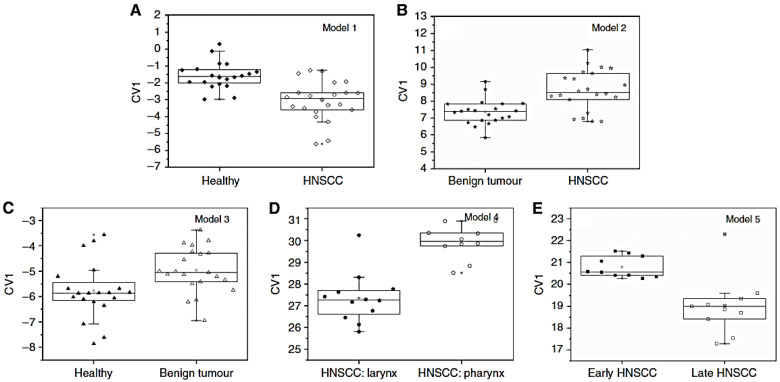
Predictive DFA models for distinguishing: (**A**) HNSCC patients from healthy (tumour-free) subjects, (**B**) HNSCC from benign tumour patients, (**C**) benign tumour patients from healthy subjects, (**D**) larynx malignancy from pharynx malignancy and (**E**) early HNSCC from late HNSCC. Reproduced with permission from [57].

Alveolar exhaled breath samples have been collected from 62 volunteers (22 HNSCC patients, 21 patients with benign tumours, and 19 healthy controls patients). The data obtained by the e-nose and analysed with DFA show that the CNTs-based sensors array is able to discriminate between different patients’ subclasses (Figure 12): healthy vs. HNSCC, benign tumour vs. HNSCC, healthy vs. benign tumour, HNSCC larynx vs. HNSCC pharynx, and early vs. late HNSCC. Accuracy is found between 73% and 95%, the sensitivity range is 71–100%, while specificity is between 75% and 91%. The lowest value is always obtained for healthy vs. benign tumour discrimination, while the best performances are obtained for discrimination of early vs. late HNSCC.

Discrimination between Alzheimer’s (AD) and Parkinson’s (PD) disease and healthy control patients using a CNTs-based e-nose is reported by Tisch et al. [58].

In detail, the DFA analysis demonstrates that the electronic nose can clearly distinguish AD from PD patients, AD from healthy patients, PD from healthy patients, and AD from PD states, with an accuracy of 84%, 85% and 78%, respectively. The use of an e-nose could represent a non-invasive screening tool for high-risk populations also in neurodegenerative disease diagnostic.

**Figure 13:**
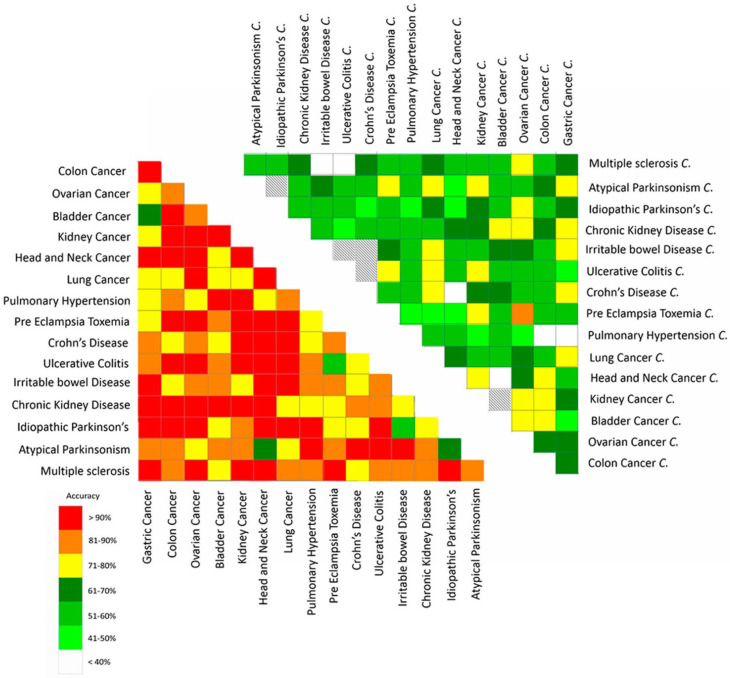
Graphical presentation of the accuracy of the binary DFA classifiers. Each box represents the accuracy achieved in a blind validation of each pair of subject groups. The left heat map gives the results of comparisons between groups of patients, whereas the graph on the right gives the results of the same classifiers applied to the corresponding control groups. The average accuracy was 86% for all disease classifiers (**left graph**) and 58% for the corresponding control groups (**right graph**). The letter “C” beside each disease named in the right figure means the “control” group relates to that specific disease. Reproduced with permission from [59].

Finally, Nakhleh et al. [59] investigated 17 different diseases: lung cancer (LC), colorectal cancer (CRC), head and neck cancer (HNC), ovarian cancer (OC), bladder cancer (BC), prostate cancer (PC), kidney cancer (KC), gastric cancer (GC), Crohn’s disease (CD), ulcerative colitis (UC), irritable bowel syndrome (IBS), idiopathic Parkinson’s (IPD), atypical Parkinsonism (PDISM), multiple sclerosis (MS), pulmonary arterial hypertension (PAH), pre-eclampsia (PET), and chronic kidney disease (CKD). The e-nose has been exposed to alveolar exhaled breath collected from 813 sick patients and 591 healthy control patients. Of note, a healthy control group is associated with each pathology. Breath sampling has been performed in nine clinic centres worldwide, following the same procedure in order to assure that the samples are comparable. To perform the DFA, three parameters have been acquired for almost all the 20 sensors in the array: relative resistance change at the end of the exposure and at the middle, and area under the curve, obtaining 59 different parameters for each breath exposure. The analysis, as shown in Figure 13, has been conducted by combining two different diseases each time, and the control patients corresponding to each disease, in order to prove that the discrimination is achieved due to the disease, and it is not influenced by any bias such as geography or methodology.

The average accuracy was 86% for all disease comparison (Figure 13—left graph) and 58% for the corresponding control groups (Figure 13—right graph). The average difference between the accuracy of the sick groups and the corresponding control groups is always sufficient to rule out a correspondence between the two classes or a bias in the data analysis related to the geography or methodology for data sampling. This difference is of course confirmed by comparing each single accuracy for the two disease classifications and the corresponding control patients. For instance, considering discrimination between pre-eclampsia and ovarian cancer, the accuracy of the classification for the sick patients is 100%, while 86% is the accuracy for the healthy control patients’ classification, which is the highest accuracy obtained for the control groups. The lowest accuracy for disease discrimination is 50% and it has been obtained for discrimination between pre-eclampsia and ulcerative colitis, whereas the corresponding accuracy for control patients is 79%.

The work proposed by Nakhleh et al. [59] is actually the study involving the largest number of patients and the highest number of tested diseases, involving more than one research centre to collect breath samples. This study could represent a starting point to develop an international database for breath samples collected with the same methodology and available for discrimination of a variety of pathologies.

## 4. Conclusions

We proposed a review on the current trends in the development of chemiresistors electronic noses using carbon nanotubes for breath analysis applications. After an introduction on the topic, we briefly focus on the functionalization on CNTs, breath sampling procedures, statistical data analysis, targeted diseases and characteristics of the population involved in the different studies. The main part of the review is dedicated to the electronic noses based on CNTs for breathomics reported in literature. Among the 26 articles found, 20 of them present electronic noses completely based on CNTs-sensors, while 6 papers report on electronic noses that couple together CNTs-sensors and NPs-based sensors. We then detail the aim of the articles, i.e., biomarker investigation, exposures to simulated exhaled breath, exposures to animal or human breath, and disclosing the potentiality of using CNTs-based e-noses in diagnostic and screening medicine. At the moment, there are few reports [35,41,45,48,52] that try to relate the materials’ properties and functionalization strategies with the overall e-nose capability to discriminate diseases. In addition, differences among sensors in the response to specific biomarkers are not always addressed. This indicates that there is room to further investigate this issue in future research on the topic. This may lead to even better performing devices, provided that the basic chemical and physical interactions of biomarkers with the sensors surface are properly recognized.

## Figures and Tables

**Table 1:** Method, type of breath, and breath container for the analysis with CNTs-based e-nose.

Online/Offline Measurements with e-Nose	Type of Breath	Bag/Container	Reference
Online	Alveolar	-	[49]
Offline	Alveolar	Mylar	[50]
Offline	Total	Glass vials	[51]
Online	Total	PTFE	[52]
Online	Total	PTFE	[53]
Offline	Alveolar	Mylar	[54]
Offline	Alveolar	GaSample	[55]
Offline	Alveolar	Tedlar	[56]
Offline	Alveolar	Mylar	[57]
Offline	Alveolar	Mylar	[58]
Offline	Alveolar	Mylar	[59]

**Table 2:** Gender, average age and smoking habits of the volunteers enrolled in each study. N.A. = data not available. * Data reported in [86].

Reference	Class	Gender (M:F)	Average Age	Smoker
[50]	Sick	13:21	N.A.	41%
	Healthy control	6:11	N.A.	41%
[51]	Sick	5:0	60–69	N.A.
	Healthy	5:0	35–60	N.A.
[52]	Sick	7:5	71 ± 6	83%
	Healthy	5:4	45 ± 15	12%
[53]	Sick	2:5	65 ± 17	29% *
	Healthy	2:2	34 ± 12	0% *
[54]	LC	31:8	62 ± 11	18%
[55]	OLGIM	102:223	59 ± 14	14%
	PUD	34:19	53 ± 15	45%
	GC	77:22	63 ± 13	29%
	Dysplasia	4:3	73 ± 8	14%
[56]	GC	28:9	58 ± 9	41%
	Non-malignant condition	23:9	51 ± 14	44%
	Less severe condition	30:31	51 ± 9	21%
[57]	HNSCC	19:3	62 ± 12	59%
	Benign tumour	14:7	55 ± 14	57%
	Healthy	6:14	50 ± 12	25%
[58]	AD	7:8	68 ± 10	N.A.
	PD	17:13	62 ± 10	N.A.
	Healthy	5:7	61 ± 7	N.A.
[59]	LC	23:22	67 ± 9	98%
	LC-control	12:11	56 ± 14	52%
	CRC	42:29	66 ± 10	11%
	CRC-control	67:22	60 ± 14	13%
	HNC	19:3	62 ± 12	59%
	HNC-control	6:13	50 ± 12	25%
	OC	0:48	51 ± 11	0%
	OC-control	0:48	47 ± 9	0%
	BC	68:5	69 ± 11	68%
	PC	11:0	66 ± 8	45%
	PC-control	31:4	66 ± 12	71%
	KC	22:11	65 ± 13	45%
	GC	57:42	63 ± 12	27%
	GC-control	55:100	57 ± 15	15%
	CD	23:18	38 ± 12	50%
	UC	20:17	41 ± 16	43%
	UC-control	28:16	41 ± 2	15%
	IBS	8:19	38 ± 13	30%
	IPD	23:21	65 ± 14	15%
	PDISM	7:9	67 ± 8	35%
	PDISM-control	19:18	62 ± 12	24%
	MS	42:76	38 ± 10	32%
	MS-control	17:27	39 ± 11	34%
	PAH	6:16	48 ± 12	54%
	PAH-control	10:13	38 ± 8	43%
	PET	0:24	30 ± 6	0%
	PET-control	0:47	29 ± 4	0%
	CKD	52:30	65 ± 12	64%
	CKD-control	12:15	46 ± 2	40%

**Table 3:** Number and type of the sensors in the electronic nose based on CNTs, number and type of biomarkers investigated, target disease, and type of data analysis. # = number.

# and Type of Sensor	# and Type of Biomarkers	Target Disease	Type of Data Analysis	Reference
5: Metal/metal oxide NPs decoreated SWCNTs	9: Ammonia, nitrogen dioxide, acetone, ethanol, 2-propanol, benzene, sodium hypochlorite, hydrogen sulfide, water	-	PCA	[34]
6: SPEEK nanocomposites based on hybrid nanocarbons	7: Ethanol, methanol, propanol, acetone, butanone, toluene, and benzene	Lung cancer	PCA	[35]
3: COOH-MWCNT functionalized with POSS	9: Acetone, butanone, propanol, ethanol, toluene, cyclohexane, pentene, methanol, water	Lung cancer, Diabetes, Malignant pleural mesothelioma	PCA	[36]
4: Metal NPs decorated CNTs	4: NH_3_, Ethanol, CO, CO_2_	-	PCA	[37]
10: SWCNTs−Metalloporphyrin	15: Pentane, Hexane, cyclohexane, acetone, MEK, 3-pentanone, methanol, ethanol, isopropanol, p-exylene, mephedrone, PhH, butylamine, DIPA, TEA	-	PCA	[38]
6: Surfactant-CNTs	6: Water, methanol, ethanol, toluene, acetone, chloroform	Lung cancer	PCA	[39]
6: CNTs conductive polymer nanocomposites	18: Water, ethanol, methanol, acetone, propanol, isopropanol, 2-butanone, chloroform, toluene, benzene, styrene, cyclohexane, o-xylene, n-pentane, n-decane, 1,2,4-trimethyl benzene, isoprene, (2-methyl-1,3-butadiene), 1-hexene	Lung cancer	PCA	[40]
6: Polymer coated CNTs	9: Chloroform, buthanol, THF, DCM, Acetone, isopropanol, toluene, water, ethanol	Lung cancer	PCA	[41]
5: CNTs conductive polymer nanocomposites	9: Dichloromethane, heptane, isopropanol, methanol, tetrahydrofuran, ethanol, toluene, cyclohexane, water	Lung cancer	PCA	[42]
8: Organic functionalized MWCNTs	20: Dodecane, octane, decane, trimethyl benzene, xylanes, toluene, benzene, chlorobenzene, dichloromethane, dihexyl ether, dibutyl ether, 2-decadone, cyclohexanone, methylethyl ketone, 1-octanol, 1-pentol, 1-butanol, ethanol	-	PCA	[43]
8: Polycyclic Aromatic Hydrocarbons/SWCNT	3: octane, ethyl benzene, ethanol	-	DFA	[44]
4: CNT/hexa-peri-hexabenzocoronene bilayers	5: Decane, octane, hexane, ethanol, water	Cancer	PCA	[45]
4: Metal NPs decorated CNTs	4: Benzene, ethylene, CO, NO_2_	-	LDA	[46]

**Table 4:** Number and type of sensors in the electronic nose based on CNTs, target disease, type of data analysis, and number of patients involved in the study. # = number.

# and Type of Sensor	Target Disease	Type of Data Analysis	# of Patients	Reference
4: Bilayers of polycyclic aromatic hydrocarbons and SWCNT	Multiple sclerosis	DFA	51 17 healthy + 34 sick	[50]
8: Polymer/SWCNTs composite	Liver Cancer	PCA	10 5 healthy + 5 sick	[51]
8: SWCNTs-organic semiconductor layers	COPD	PCA/SVM	21 9 healthy + 12 sick	[52]
8: SWCNTs-organic semiconductor layers	COPD	PCA/SVM/LDA	50 30 sick + 20 healthy	[53]

**Table 5:** Number of the sensors in the electronic nose based on NPs and CNTs, the target disease, and the number of patients tested. # = number, N.A. = not available.

# of Sensors	Target Disease	# of Patients	Reference
40: composition N.A.	Lung cancer	39, all sick	[54]
8: 6 Au NPs + 2 CNTs	Precancerous gastric lesion, peptic ulcers (PUD) and gastric cancer (GC)	484, all sick (325 precancerous lesion, 53 PUD, 99 GC, 7 dysplasia)	[55]
14: 10 Au NPs + 4 CNTs	Gastric cancer and benign gastric condition	130, all sick (37 cancer, 32 non-malignant gastric condition, 61 less severe condition)	[56]
6: 5 Au NPs + 1 CNTs	Head and Neck squamous cell carcinoma (HNSCC)	62, sick and healthy (22 HNSCC, 21 benign tumours, 19 healthy)	[57]
8: 6 Au NPs + 2 CNTs	Alzheimer’s (AD) and Parkinson’s (PD) disease	57, sick and healthy (15 AD, 30 PD, 12 healthy)	[58]
20: 17 Au NPs + 3 CNTs	Lung cancer (LC), colorectal cancer (CRC), head and neck cancer (HNC), ovarian cancer (OC), bladder cancer (BC), prostate cancer (PC), kidney cancer (KC), gastric cancer (GC), Crohn’s disease (CD), ulcerative colitis (UC), irritable bowel syndrome (IBS), idiopathic Parkinson’s (IPD), atypical Parkinsonism (PDISM), multiple sclerosis (MS), pulmonary arterial hypertension (PAH), pre-eclampsia (PET), chronic kidney disease (CKD), healthy	1404, sick and healthy (45 LC, 71 CRC, 22 HCN, 48 OC, 73 BC, 11 PC, 33 KC, 99 GC, 41 CD, 37 UC, 27 IBS, 44 IPD, 16 PIDSM, 118 MS, 22 PAH, 24 PET, 82 CKD, 591 healthy	[59]

## Data Availability

Not applicable.
